# Understanding astrocyte differentiation: Clinical relevance, technical challenges, and new opportunities in the omics era

**DOI:** 10.1002/wsbm.1557

**Published:** 2022-05-12

**Authors:** Michael Lattke, Francois Guillemot

**Affiliations:** ^1^ Neural Stem Cell Biology Laboratory The Francis Crick Institute London UK; ^2^ Present address: Department for Brain Sciences Imperial College London London UK

**Keywords:** astrocytes, cell differentiation, epigenomics, neural development, neurological disorders, neural repair/regeneration, transcriptomics

## Abstract

Astrocytes are a major type of glial cells that have essential functions in development and homeostasis of the central nervous system (CNS). Immature astrocytes in the developing CNS support neuronal maturation and possess neural‐stem‐cell‐like properties. Mature astrocytes partially lose these functions but gain new functions essential for adult CNS homeostasis. In pathological conditions, astrocytes become “reactive”, which disrupts their mature homeostatic functions and reactivates some immature astrocyte‐like properties, suggesting a partial reversal of astrocyte maturation. The loss of homeostatic astrocyte functions contributes to the pathogenesis of various neurological conditions, and therefore activating maturation‐promoting mechanisms may be a promising therapeutic strategy to restore homeostasis. Manipulating the mechanisms underlying astrocyte maturation might also allow to facilitate CNS regeneration by enhancing developmental functions of adult astrocytes. However, such therapeutic strategies are still some distance away because of our limited understanding of astrocyte differentiation and maturation, due to biological and technical challenges, including the high degree of similarity of astrocytes with neural stem cells and the shortcomings of astrocyte markers. Current advances in systems biology have a huge potential to overcome these challenges. Recent transcriptomic analyses have already revealed new astrocyte markers and new regulators of astrocyte differentiation. However, the epigenomic changes that presumably occur during astrocyte differentiation remain an important, largely unexplored area for future research. Emerging technologies such as CRISPR/Cas9‐based functional screens will further improve our understanding of the mechanisms underlying astrocyte differentiation. This may open up new clinical approaches to restore homeostasis in neurological disorders and/or promote CNS regeneration.

This article is categorized under:

Neurological Diseases > Genetics/Genomics/Epigenetics
Neurological Diseases > Stem Cells and Development
Neurological Diseases > Molecular and Cellular Physiology

## Graphical abstract

Astrocytes have important functions supporting brain development and homeostasis. Systems biology offers new opportunities to identify mechanisms driving astrocyte differentiation and maturation, which may allow the therapeutic modulation of astrocytic functions to promote homeostasis or regeneration.

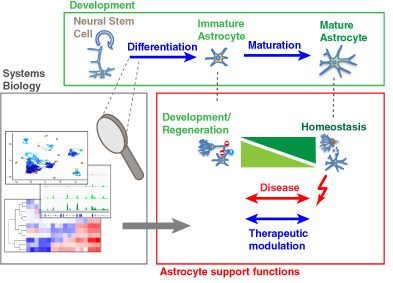

## 1 INTRODUCTION

Astrocytes are a major type of glial cells of the central nervous system (CNS). Astrocytes are generated by neural stem cells (NSCs) after they have produced neurons, during late embryogenesis and early postnatal life. While immature astrocytes in the developing CNS support neuronal maturation and possess NSC‐like properties, mature astrocytes in the adult CNS are essential to maintain tissue homeostasis and modulate the activity of neuronal networks (Akdemir et al., 2020; Dallerac et al., 2018; Laywell et al., 2000; Molofsky & Deneen, 2015). In pathological conditions, astrocytes undergo dramatic morphological and functional changes, a process called “astrocyte reactivity” or “astrogliosis.” In these conditions, astrocytes gain immune cell‐like inflammatory functions and their mature homeostatic functions are often disrupted (Escartin et al., 2021; Lattke & Wirth, 2017; Sofroniew, 2020; Sofroniew & Vinters, 2010). Reactive astrocytes also regain to some extent the functions and NSC‐like properties of immature astrocytes (Anderson et al., 2016; Buffo et al., 2008; Magnusson et al., 2014), suggesting that astrocyte reactivity partially reverts astrocyte maturation. While the disruption of homeostatic astrocyte functions is thought to contribute to the pathogenesis of many neurological disorders (Escartin et al., 2021; Lattke & Wirth, 2017; Sofroniew, 2020; Sofroniew & Vinters, 2010), the reactivation of immature astrocyte properties might support the limited regenerative potential of the CNS (Anderson et al., 2016; Buffo et al., 2008; Magnusson et al., 2014). Beside changes in properties reflecting developmental maturation and pathological reactivity, astrocytes also display morphological and molecular regional heterogeneities, whose functional relevance is not well understood yet.

Because of the diverse functions of astrocytes, elucidating the mechanisms that confer astrocytes their identity and mature features, is of crucial importance for the understanding of brain development and function, as well as for the understanding of the pathogenesis of neurological disorders and the lack of regenerative capacity of the CNS. This is highly relevant for clinical medicine, as neurological disorders and CNS injuries have devastating consequences for individuals, pose a major burden to healthcare systems, and effective therapies are often lacking. However, progress in understanding astrocyte development and function has been slow compared to the rapid progress made in characterizing neuronal development, and this for various reasons: first, neuroscience has traditionally been centered on neuronal biology, therefore research into astrocytes has been neglected for a long time. As a result, tools for studying astrocytes have long been underdeveloped. Furthermore, understanding astrocyte development and function is complicated by their biological characteristics. In particular, astrocytes are very similar to NSCs, which makes it difficult to distinguish the two cell types using classical marker‐based approaches (Gotz et al., 2015) and complicates the interpretation of many studies assessing astrocyte development. Only the development of large‐scale systems biology approaches within the last 15 years, in particular high‐throughput sequencing technologies, has allowed scientists to gain a progressively more detailed and accurate understanding of the molecular identity and developmental trajectory of astrocytes. This has already led to major progress in understanding astrocyte biology, although many of the novel and powerful systems biology technologies have not yet been applied to this emerging field of research.

In this review, we will provide a broad overview of the essential functions of astrocytes throughout brain development and homeostasis, and of their relevance in the context of CNS disorders and injury. We will then present the challenges that have long slowed down progress in understanding astrocyte development and function on a molecular level, and we will review the progress that has been achieved so far, both before and during the “omics era”. Finally, we will discuss the future directions and opportunities arising from the advent of systems biology approaches for understanding astrocyte development, and the implications that this research may have for the development of new treatment strategies for neurological disorders and for regenerative medicine.

## 2 ASTROCYTE DEVELOPMENT AND FUNCTION IN HEALTH AND DISEASE

### 2.1 Overview of astrocyte development

**FIGURE 1:**
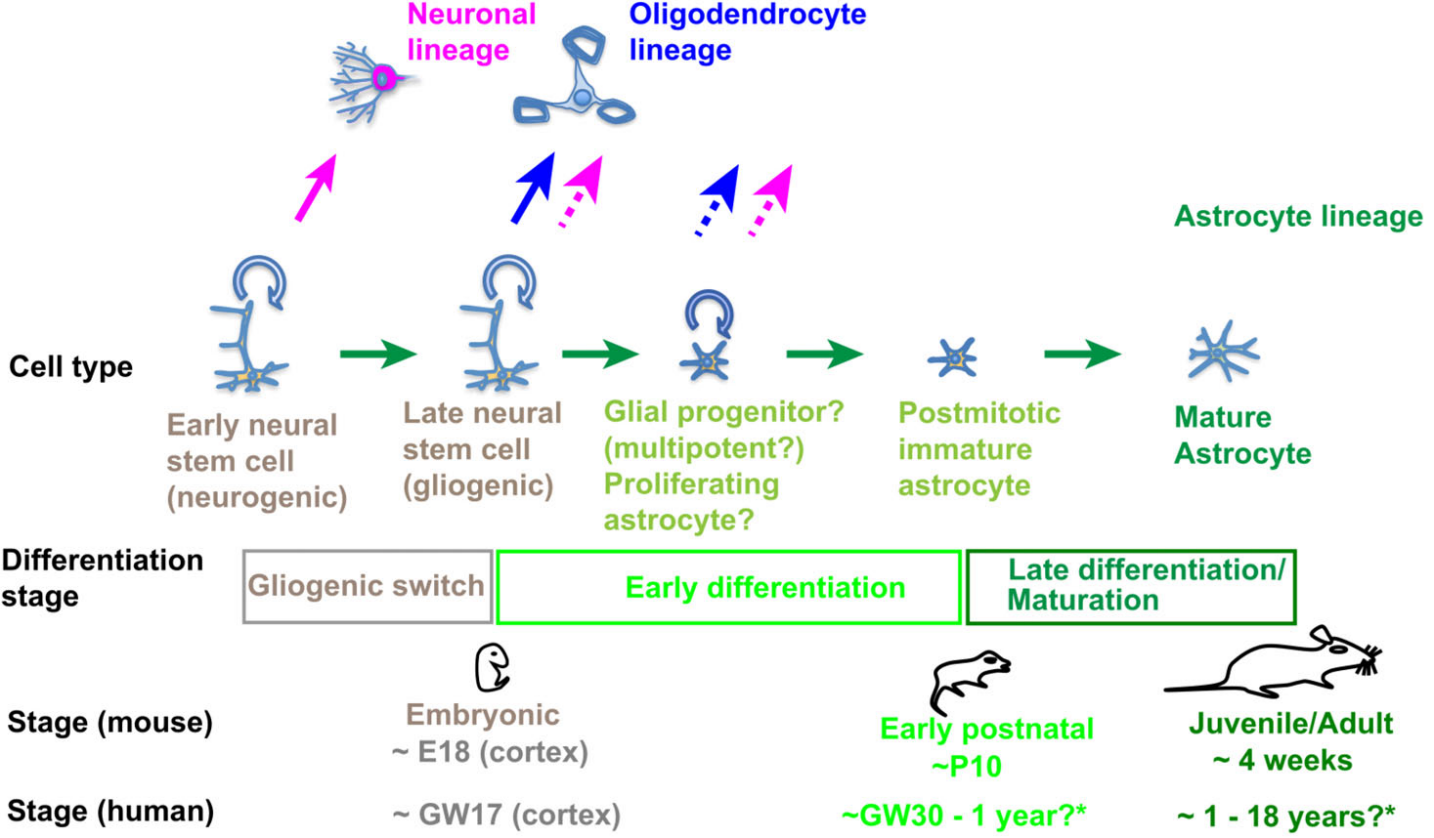
Overview of astrocyte development. Neural stem cells (NSCs) during embryonic stages generate neurons before the “gliogenic switch” occurs, after which they predominantly generate oligodendrocyte and astrocyte lineage cells. Through incompletely characterized proliferative and postmitotic immature stages, astrocytes differentiate and mature during late brain development development, in mice between perinatal stages and around 4 weeks of age. *Estimated stages in humans with different approaches (see text)

During early neural development in mammals, regional patterning events generate locally distinct subpopulations of neural stem/progenitor cells (NSCs/NPCs) derived from the embryonic neuroectoderm (Bayraktar et al., 2014; Kriegstein & Alvarez‐Buylla, 2009; Ortiz‐Alvarez & Spassky, 2020; Solanelles‐Farre & Telley, 2020). These NPCs show similarities to astroglial cells, as described in detail in the following sections, and are therefore also called radial glia. They reside in the germinal zones of the central nervous system (CNS), the ventricular zones adjacent to the cerebral ventricles, and their radial processes span the developing brain parenchyma. Initially, during mid to late embryonic development, radial glia generate the majority of neurons before they acquire the ability to generate glial cells, a transition known as the “gliogenic switch” (Figure 1). Gliogenic NPCs in the ventricular zone retain the capacity to generate both astrocytes and oligodendrocytes and subsequently generate glial precursor cells that are thought to be restricted to an astrocytic or oligodendrocytic fate and migrate into the brain parenchyma. Local glial precursors continue to proliferate at postnatal stages before differentiating into astrocytes or oligodendrocytes and undergoing extensive functional maturation. This general model of glial development is widely accepted and has been recently been described in more detail in several excellent reviews (Akdemir et al., 2020; Bayraktar et al., 2014; Molofsky & Deneen, 2015).

The time course of astrocytic lineage development is relatively well characterized in the mouse cortex, but not in other brain regions and species. In mice, the gliogenic switch occurs around embryonic day 18 (E18) in the cortex, and the generation of cortical astrocytes is completed around postnatal day 10 (P10). After this point, maturation of astrocytes is thought to continue until around 3–4 weeks of age (Akdemir et al., 2020; Yang et al., 2013). In other brain regions, this timing might differ considerably, in line with the overall developmental timelines of these regions. For example, the gliogenic switch in the murine spinal cord occurs around E12 (Akdemir et al., 2020; Yang et al., 2013). In humans, gliogenesis in the cortex is thought to start around gestational week 17 (GW17) (Clancy et al., 2001; Semple et al., 2013; Zhang et al., 2016), while reported estimated times for completion of gliogenesis and astrocyte maturation vary widely. While it has been reported that cells with clear astroglial features appear only around birth and gliogenesis continues until around 1 year postnatally, transcriptomic analyses found adult expression levels of most mature astrocyte markers already around 6 to 12 months of age, suggesting that most astrocytes are already mature at these stages (Clancy et al., 2001; Semple et al., 2013; Zhang et al., 2016). On the other hand, interspecies comparisons of brain development would suggest that the generation of immature astrocytes might be largely complete around GW30‐40, while maturation might continue until ages of 12–18 years, assuming astrocyte development occurs at similar stages of overall brain development in mice and humans (Clancy et al., 2001; Semple et al., 2013).

Not only the time course, but also the identity and properties of intermediate differentiation stages of the astrocyte lineage remain ill‐defined, and it is not well understood how the heterogeneity and functional diversity of astrocytes in brain development and homeostasis are established, as discussed in detail in Sections 3 and 4.

### 2.2 Functions of immature astrocytes in brain development

**FIGURE 2:**
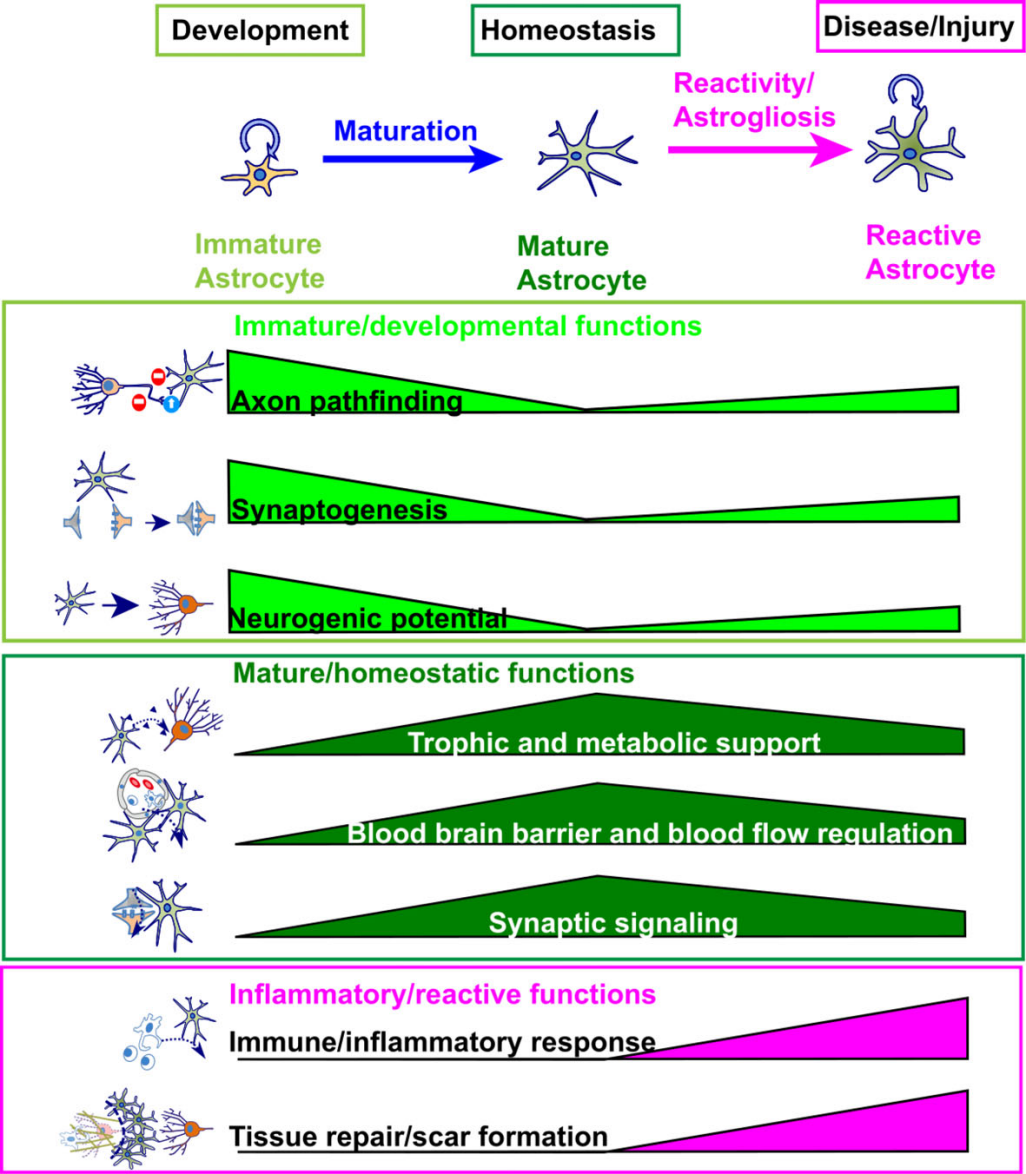
Astrocyte functions in development, homeostasis and disease. Immature astrocytes in the developing brain support neuronal maturation and possess a NSC‐like neurogenic potential. During maturation these functions become limited, while new functions regulating adult brain homeostasis and neuronal signaling emerge. In pathological conditions astrocytes become reactive, which leads to a partial reversal of functional maturation and a gain of immune cell‐like functions

During late stages of brain development, astrocytes serve multiple critical functions in supporting the wiring and maturation of neurons (Figure 2). Pioneering studies showed that in vitro, astrocytes promote neurite outgrowth and synapse formation of co‐cultured neurons (Noble et al., 1984; Pfrieger & Barres, 1997), suggesting that astrocytes promote the formation and maturation of neuronal circuits. Over the last 20 years, the function of astrocytes in promoting synapse formation has been extensively studied, confirming the synaptogenic roles of astrocytes in vivo, and identifying a number of astrocytic proteins involved in this function, such as Thrombospondins, Hevin and Neuroligins (Christopherson et al., 2005; Kucukdereli et al., 2011; Stogsdill et al., 2017). In addition, astrocytes have also been found to contribute to the developmental elimination of synapses, a process essential for the refinement of neuronal circuits during experience‐dependent brain maturation (Chung et al., 2013). Much less well understood is the role of astrocytes in axon growth and guidance in vivo. Astrocytes produce a large number of extracellular matrix proteins and express cell adhesion molecules, which can either promote or inhibit axonal growth (Dallerac et al., 2018; Fawcett, 2020). However, how this modulates axonal growth during normal development in vivo has not been studied extensively. An elegant study showed nevertheless that expression of the secreted guidance protein Sema3a by a specific subpopulation of astrocytes in the developing spinal cord is required for appropriate growth of motor axons (Molofsky et al., 2014), demonstrating that astrocytes may indeed control axonal growth in vivo.

Beside their role in supporting neuronal maturation, astrocytes are thought to contribute to brain angiogenesis and the establishment of the blood–brain‐barrier (BBB) (Araya et al., 2008; Lee et al., 2003), although this function is still debated (Daneman & Prat, 2015; Zhao et al., 2015). For example, studies have shown that astrocytes induce BBB‐like properties in endothelial cells in vitro and in transplantation models, and a recent study demonstrated that genetic ablation of astrocytes disrupts the BBB (Heithoff et al., 2021; Janzer & Raff, 1987; Siddharthan et al., 2007). On the other hand, an earlier study using a similar astrocyte ablation in vivo did find such defects, and moreover, initial BBB‐like barrier properties of the cerebral vasculature are established at embryonic stages preceding astrogliogenesis in mice (Daneman et al., 2010; Tsai et al., 2012).

Finally, astrocytes may control developmental myelination, as they have been shown to provide oligodendrocytes with lipids required for the myelination of axons (Camargo et al., 2017), which is crucial for appropriate propagation of action potentials and the function of neuronal networks.

### 2.3 Functions of mature astrocytes in the adult brain

As astrocytes become mature during the first 3–4 postnatal weeks in mice (Yang et al., 2013), they undergo prominent functional changes, from supporting brain development to supporting brain homeostasis and modulating neuronal communication (Figure 2). As astrocytes become mature, their neuronal development‐supporting functions become more limited. For example, astrocytes are thought to switch from producing extracellular matrix proteins that support axonal outgrowth to producing proteins that inhibit axonal outgrowth, which has been proposed to contribute to the limited regenerative capacity of the adult CNS (Dallerac et al., 2018; Jones & Bouvier, 2014).

On the other hand, maturing astrocytes gain new functions, for example in the control of the activity‐dependent local supply of neurons with metabolites and oxygen. As energy consumption of the brain is very variable depending on the local activity of neuronal networks, these astrocytic functions are essential for neuronal survival and normal brain function. Astrocytes interact closely with both neurons and vascular cells (endothelial cells and pericytes), allowing them to sense local neuronal activity and relay this information to vascular cells, which in response modulate the metabolite supply to neurons by regulating the local blood flow and the transport of metabolites through the blood–brain barrier (Attwell et al., 2010; Daneman & Prat, 2015). Astrocytes subsequently transform these metabolites into molecules that are easily usable by neurons as energy metabolites and/or neurotransmitter precursors, most importantly lactate and glutamine (Magistretti & Allaman, 2018; Schousboe, 2019). Furthermore, astrocytes can also store limited amounts of glucose as glycogen, a quickly mobilizable energy storage, which allows astrocytes to serve as local buffers for fluctuations in energy supply (Alberini et al., 2018).

Beside their metabolic support functions, astrocytes actively modulate the activity of neuronal networks, constituting an important component of the “computational system” of the brain. Multiple well characterized mechanisms allow astrocytes to prevent excessive neuronal activity (Dallerac et al., 2018). Through potassium channels, most prominently Kir4.1 (*Kcnj10*), astrocytes take up potassium released upon neuronal excitation. Through two glutamate transporters, GLAST (EAAT1, *Slc1a3*) and GLT‐1 (EAAT2, *Slc1a2*), and transporters for other neurotransmitters, astrocytes take up neurotransmitters released by the pre‐synaptic compartment. In addition, astrocytes can also modulate neuronal activity by the release of so‐called gliotransmitters, including glutamate, GABA, D‐serine, and ATP and other adenosine derivatives (Dallerac et al., 2018; Henneberger et al., 2010; Illes et al., 2019; Ishibashi et al., 2019; Yoon et al., 2014). Finally, astrocytes also modulate the long‐term activity of neuronal networks by secreting neurotrophic factors such as Bdnf (Fernandez‐Garcia et al., 2020) and other secreted and structural molecules required to maintain and remodel synapses (Christopherson et al., 2005; Kucukdereli et al., 2011; Stogsdill et al., 2017).

### 2.4 Astrocyte reactivity ‐ functional changes in pathology

Another set of astrocyte functions that are crucial for maintaining brain homeostasis, are their prominent responses to injury and infections. In a vast range of pathological conditions, including acute injury, stroke, epilepsy, multiple sclerosis, and chronic neurodegenerative disorders such as Alzheimer's disease and amyotrophic lateral sclerosis (ALS), astrocytes show prominent and heterogenous morphological and functional alterations, which are usually referred to as “astrocyte reactivity” or “astrogliosis” (Figure 2) (Allaman et al., 2011; Sofroniew & Vinters, 2010). In these conditions, astrocytes become hypertrophic and upregulate the intermediate filament and classical astrocyte marker GFAP, as well as various inflammatory mediators and effector molecules and extracellular matrix proteins (Escartin et al., 2021; Lattke & Wirth, 2017; Sofroniew, 2020; Sofroniew & Vinters, 2010). Furthermore, reactivity can interfere with mature astrocyte homeostatic functions, such as glutamate uptake, potassium buffering, metabolic support and blood–brain barrier maintenance, which is thought to contribute to the pathogenesis of various neurological disorders, such as epilepsy or ALS (Verhoog et al., 2020; Yamanaka et al., 2008). These changes have led to a traditional view of astrocyte reactivity as a stereotypical response to pathological conditions, which is usually considered detrimental as it can result in excessive inflammatory responses and can impair regeneration by promoting scar formation in injury and chronic neurodegenerative disorders (Sofroniew, 2009). However, in recent years a more nuanced view of astrocyte reactivity has emerged, suggesting that there are distinct states of astrocyte reactivity which can be either detrimental or protective depending on the context (Escartin et al., 2021; Liddelow et al., 2017; Zamanian et al., 2012). For example, a key study has shown that reactive astrocytes in the glial scar formed after spinal cord injury do not impair axonal regeneration, as had been long assumed, but actually promote axonal regeneration induced by a preconditioning lesion (Anderson et al., 2016). Interestingly, in some conditions reactive astrocytes can re‐enter the cell cycle and dedifferentiate to more immature and potentially regeneration‐promoting states. In particular after acute injury or Notch inhibition, at least some reactive astrocytes have even been shown to revert to a neural‐stem‐cell‐like state and generate limited numbers of new neurons in vitro and in vivo (Buffo et al., 2008; Laywell et al., 2000; Magnusson et al., 2014; Nato et al., 2015; Sirko et al., 2013).

The plasticity of astrocytes in pathologies is also thought to be a critical factor for the development of many brain tumors, including glioblastoma, one of the most devastating types of cancer. Many brain tumors contain large numbers of cells with astroglial characteristics, and at least in mouse models, glial progenitors and even mature astrocytes can serve as cells of origin for glioblastoma‐like tumors, by reactivating stem‐cell‐like properties upon oncogenic transformation with viral vectors (Friedmann‐Morvinski et al., 2012). Furthermore, both astrocyte‐like tumor cells and tumor‐associated astrocytes in the surrounding brain tissue adopt a reactive state which is thought to facilitate tumor growth via secretion of inflammatory cytokines and growth factors (Zhang et al., 2020).

Beside the broad functional changes occuring in astrocytes when they become reactive, and that are thought to modify the pathogenesis of a wide range of disorders, as outlined above, selective defects in specific astrocyte functions are thought to underly a number of genetic neurodevelopmental disorders. The prototypical “astrogliopathy” is Alexander disease, caused by mutations in the astrocytic intermediate filament Glial Fibrillary Acidic Protein (GFAP), which result in general astrocyte dysfunction, resulting in various symptoms including epileptic seizures, macrocephaly and cognitive and motor delay (Sosunov et al., 2018). Other examples are Rett syndrome, in which disruptions of developmental support functions of astrocytes seem to contribute to dendritic and synaptic pathology (Lioy et al., 2011), and epilepsy syndromes caused by mutations in genes required for the homeostatic modulation of synaptic signaling by astrocytes, such as the glutamate transporter SLC1A2 (GLT‐1) or the glutamate‐metabolizing enzyme glutamine synthetase (GLUL) (Eid et al., 2019; Epi, 2016).

## 3 CHALLENGES IN STUDYING ASTROCYTE DEVELOPMENT

Despite the important functions of astrocytes that we have reviewed above, many aspects of their biology remain poorly understood, including in particular the molecular mechanisms that underpin their development and function, because of unique technical and biological challenges that complicate research on astrocytes.

### 3.1 Identifying astrocytes: The GFAP problem

One issue which has hampered progress in the astrocyte field and complicates the interpretation of many studies, is the traditional reliance on the intermediate filament protein Glial Fibrillary Acidic Protein (GFAP) as main or only marker to identify astrocytes. GFAP is easily detectable by immunolabeling, it is not expressed by the vast majority of other cells in the brain, including neurons, oligodendrocytes, oligodendrocyte precursors, microglia, endothelial cells and pericytes, and its expression gives some information about the normal or reactive morphology of astrocytes. Despite the long‐recognized shortcomings of this marker, numerous studies have used expression and regulation of GFAP as a surrogate for astrocyte development and function, which is a highly problematic generalization.

The main problems are that not all astrocytes do express GFAP, and astrocytes are not the only cells that do. In particular, most astrocytes in the cortical gray matter express GFAP at levels that are barely or not detectable by immunolabeling and related techniques (Cahoy et al., 2008), while developmental radial glia and adult neural stem cells do express variable levels of GFAP (Doetsch et al., 1999; Gotz et al., 2015). Also, GFAP expression is highly regulated in various conditions, in particular in pathology. Moreover, GFAP is predominantly expressed in the main cell processes, so GFAP labeling is not well suited either to identify astroglial cell bodies, or to visualize the much more complex overall cell volume. Overall, this can easily lead to wrong conclusions about changes in astrocyte numbers and morphology (Escartin et al., 2021).

One of the few alternative astrocyte markers that have been used alongside with GFAP, although less widely, is S100ß, a cytoplasmic calcium binding protein restricted to mature astrocytes, and absent from neural stem cells and immature astrocytes. Many other well‐known astrocytic proteins, such as Aqp4, Cx30, Cx43, GLAST, or GLT‐1, are not suitable for immunolabeling of astrocytes, because they decorate either the whole plasma membrane, for example, mainly the small, densely packed and complex astrocyte processes, or only very specific compartments (e.g., astrocyte endfeet at blood vessels or gap junctions), which prevents a clear and unambiguous labeling of astrocytes (Escartin et al., 2021). Furthermore, like S100ß, these markers have the disadvantage to be expressed predominantly by mature astrocytes and not by immature astrocyte populations. However, regulatory sequences for several of these markers have been used in recent years to drive expression of transgenes, which allows for a relatively selective manipulation of astrocytes and their visualization with reporter genes (Yu et al., 2020).

Overcoming some limitations of the classical markers mentioned above, several alternative markers for astrocytes have been identified in recent years and are slowly gaining more attention. In a pioneering study, Ben Barres' lab identified Aldh1l1 as an alternative broadly usable cytoplasmic astrocyte marker (Cahoy et al., 2008), and Aldh1l1 is progressively becoming more widely used for immunolabeling and transgenic approaches. Sox9 is another marker for astrocytes which has been described recently (Sun, Cornwell, et al., 2017) and, due to its nuclear localization, is particularly useful for cell number quantification and co‐staining with other nuclear factors. However, both Aldh1l1 and Sox9, along with many other astrocyte markers, are also expressed in neural stem and progenitor cells (Gotz et al., 2015; Sun, Cornwell, et al., 2017), which have very similar properties to immature astrocytes. This biological feature is singularly complicating investigations into astrocytes, as discussed below. Therefore, the careful choice of markers, dependent on the addressed question, is crucial to advance the understanding of the development of astrocytes and their plasticity in pathologies. In Table 1 we recommend markers suitable for several applications. However, we want to emphasize, as extensively discussed in this review, that all these markers have serious limitations, and that multiple markers or where possible genome‐wide approaches should be used to assess astrocyte differentiation and their plasticity in pathologies.

**TABLE 1:** Recommended markers for astrocytes for different applications (details/references see main text)

Application	Recommended markers	Limited suitablity
Labeling whole lineage, quantification of total astrocyte numbers (outside neurogenic zones)	Sox9, Sox2 (nuclear); Aldh1l1 (cytoplasm/cell body)	Glul/GS (low at early stages); Slc1a3/GLAST (only RNA); GFAP (variable expression, may miss subsets, difficult to locate cell bodies)
Labeling immature astrocytes (and progenitors/ stem cells)	Fabp7/BLBP (cytoplasm/cell body)	Nes (good for RNA, but only stains major processes, difficult to locate cell bodies); whole lineage + proliferation markers (for proliferating subsets)
Labeling mature astrocytes (not all subtypes may express each marker)	S100β (cytoplasm/cell body)	Slc1a2/GLT‐1, Gjb6/Cx30, Aqp4 (all good for RNA, but stain only peripheral processes); Glul/GS (also low expression at early stages)
Detecting major morphological changes, pathological changes in tissue	GFAP (major processes)	Nes (only in immature/reactive astrocytes), Aldh1l1 (can capture major morphological changes)
Labeling of peripheral processes	(Ideally sparse genetic labeling)	Slc1a2/GLT‐1 (perisynaptic processes), Gjb6/Cx30 (gap junctions), Aqp4 (endfeet); (all only for mature astrocytes, visualization difficult due to process density and size)
Visualizing full morphology, quantifying cell size/volume	(Requires sparse genetic labeling)	GFAP, Aldh1l1 (no or poor labeling of peripheral processes)
Distinguishing immature astrocytes and neural stem cells	(No established unique markers; Combination of markers, morphological and location criteria, or quantitative omics analyses required)	

### 3.2 Similarity between astrocytes and neural stem cells

Many astrocyte markers, including GFAP, Sox9, or Aldh1l1, are also expressed by neural stem cells. Reciprocally, many genes used traditionally as marker for neural stem and progenitor cells, such as Nestin or Sox2, are also expressed by astrocytes, in particular by immature and reactive astrocytes (Gotz et al., 2015; Sun, Cornwell, et al., 2017). Furthermore, immature and reactive astrocytes also share many functional properties with neural and progenitor stem cells, including importantly their self‐renewal and multi‐lineage potential in vitro, and in some conditions in vivo (Buffo et al., 2008; Laywell et al., 2000; Magnusson et al., 2014; Sirko et al., 2013).

The similarities between astrocytes and neural stem cells make it difficult to distinguish the two cell types, particularly at late developmental stages, which has largely prevented a detailed consensus definition of the different stages of astrocyte development. Indeed, while early neural stem/progenitor cells, which are initially producing only neurons, can be unambiguously classified as neural stem cells, the situation at later stages is less clear. After the gliogenic switch, NSCs (radial glia) start to express GFAP and predominantly generate glial cells (Gotz et al., 2015), and therefore could be regarded as glial progenitor cells. Many studies even define the onset of astrocytic differentiation of NPCs by the onset of GFAP expression, but this is inaccurate as GFAP expressing radial glia still generate oligodendrocytes and in some cases neurons. Therefore, late radial glia are usually, and correctly, still considered as NSCs (albeit mostly glial‐restricted). Proliferating, GFAP‐expressing cells with an astrocytic morphology are then found in the early postnatal mouse cortex (Ge et al., 2012). These cells seem to retain a multi‐lineage and self‐renewal potential in vitro until around postnatal day 10 in mice (Laywell et al., 2000), and could therefore also be regarded as NSCs. However their fate in vivo suggests that they likely rather represent dedicated astrocyte progenitors, equivalent to the much better characterized proliferating neuronal intermediate progenitor cells (IPCs) and oligodendrocyte precursor cells (OPCs), also found in the early postnatal cerebral cortex. The multi‐lineage potential of these astrocyte progenitors observed in vitro would then merely reflect the general plasticity of astrocytic lineage, which is also found in reactive astrocytes from the adult brain (Buffo et al., 2008).

### 3.3 Modeling astrocyte development in vitro

The exclusive reliance on GFAP as astrocyte marker and the similarities of neural stem cells and astrocytes are two issues that have also greatly complicated the investigation of astrocyte development and function in vitro. The “gold standard” for the in vitro study of astrocytes is still to use primary astrocyte cultures derived from brain tissues using variations of the method from McCarthy & de Vellis (Lange et al., 2012; McCarthy & de Vellis, 1980). In this approach, mixed cell suspensions from enzymatically dissociated early postnatal mouse or rat brain tissue are cultured in serum‐containing medium (sometimes supplemented with mitogens such as Epidermal Growth Factor [EGF] and Fibroblast Growth Factor 2 [FGF2]), which results in the rapid expansion of a population of large, flat, polygonal cells that express high levels of GFAP. These primary astrocytes support neuronal survival and neurite outgrowth, respond to inflammatory stimuli with the production of inflammatory mediators, can take up glutamate, and show other properties associated with astrocytes in vivo (Lange et al., 2012; Noble et al., 1984). However, these functional properties cannot be directly compared quantitatively with those of astrocytes in vivo, making it difficult to evaluate how similar primary cultured astrocytes are to mature astrocytes in vivo.

Primary cultured astrocytes have several limitations that limit their usefulness to model astrocyte development and function in vivo, including the two‐dimensional morphology of cultured astrocytes and the potential contamination with other cell types present in the original mixed cell population. Furthermore, the in vivo origin of these cells is unclear. While astrocytes form a large fraction of all brain cells, primary astrocyte cultures seem to derive from the clonal expansion of a relatively small number of cells, suggesting that the culture conditions select for a specific subtype or developmental stage of the large in vivo astrocyte population. Indeed, it seems likely that primary astrocytes derive from a population of proliferating neural stem cell‐like progenitor cells, which are pushed towards a GFAP‐positive astrocyte‐like phenotype by exposure to serum (Lange et al., 2012). Primary astrocytes cultured in the presence of EGF and FGF2 and in the absence of serum, return to a multipotent and proliferative state similar to that of NSCs cultured in the same conditions (Laywell et al., 2000). Conversely, NSCs cultured in the presence of serum present a GFAP‐positive astrocyte‐like phenotype similar to that of cultured primary astrocytes (Michelucci et al., 2015). This suggests that only NSC‐like astrocyte progenitors, but not postmitotic and mature astrocytes, can be cultured in conventional conditions. This would explain that very few studies have reported the culture of astrocytes derived from adult brain tissue (He et al., 2019; Sun, Hu, et al., 2017; Zarei‐Kheirabadi et al., 2020). Of note, these later preparations include brain tissue from neurogenic regions, especially the adult subventricular zone (He et al., 2019; Zarei‐Kheirabadi et al., 2020), suggesting that “adult astrocytes” might in fact be differentiated in vitro from subventricular zone NSCs. Further emphasizing their differences with mature astrocytes in the adult brain, primary astrocytes express markers of neural stem cells and immature astrocytes (e.g., Nestin) and high levels of inflammatory mediators (Foo et al., 2011; Sergent‐Tanguy et al., 2006; Zamanian et al., 2012), while they lack expression of many genes found in mature astrocytes, such as GLT‐1 or Cx30 (Hasel et al., 2017). Overall, this suggests that primary astrocytes represent an immature cell population of uncertain origin and with features of neural‐stem‐cells and reactive astrocytes, which greatly limits their usefulness to model late stages of astrocyte development or mature astrocyte function.

Over the last 20 years, a number of alternative methods to generate mature astrocytes in vitro have been described, usually involving the differentiation of cultured astrocyte‐like GFAP‐positive cells obtained from rodent and, more recently, human ESC‐derived cultures or primary NSC cultures. The vast majority of these protocols use Bone Morphogenetic Proteins (BMPs) to activate SMAD signaling, or JAK‐STAT‐pathway agonists such as CNTF or LIF, which are known potent inducers of GFAP expression (Bonaguidi et al., 2005; Chandrasekaran et al., 2016; Kleiderman et al., 2015; Krencik et al., 2011; Tiwari et al., 2018). Cells obtained with these different protocols usually show properties similar to those of cultured primary astrocytes, although sometimes a less reactive astrocyte‐like phenotype and a stellate morphology more reminiscent to that of astrocytes in vivo. However, in most cases the characterization of cells obtained in these models remains rather superficial, showing, for example, GFAP expression, “astrocyte‐like” cell morphology or isolated in vitro functional assays. Therefore, it remains unclear to which extent these in vitro‐differentiated cells resemble mature astrocytes in vivo as opposed to immature astrocytes or GFAP‐positive quiescent NSCs.

While astrocyte culture models have been useful to study molecular processes occurring during early stages of astrocyte development and to investigate individual astrocyte functions, a more thorough characterization of the cells generated in these cultures is required to determine whether they can be used to model later stages of astrocyte development or mature astrocyte functions.

## 4 DEFINING THE MOLECULAR IDENTITY AND DEVELOPMENT OF ASTROCYTES

**FIGURE 3:**
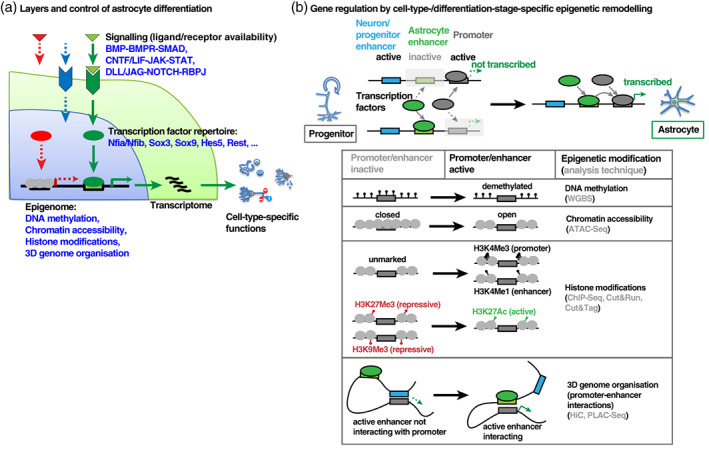
Molecular control of astrocyte identity and function. (a) The molecular identity and cell‐type‐specific functions of astrocytes are largely determined by their transcriptional profile, which is regulated on multiple levels, by extrinsic signals, transcription factors and the epigenetic state. (b) The ability of transcription factors to induce gene expression is regulated by the activity of cell type‐specific regulatory DNA elements, which is defined by multiple layers of epigenetic modifications and chromatin properties, which can be interogated by different genome‐wide analysis methods

The biological functions of a cell are determined to a large extent by the repertoire of expressed genes, which in turn is shaped by a complex interplay of extrinsic signals, transcription factors, and the epigenetic state of the cell (Figure 3). A number of studies using traditional, candidate‐focused and marker‐based approaches have resolved some of the mechanisms underlying astrocyte development and function (see Figure 3 and the following sections). However, the high level of similarity between astrocytes and NSCs, the inconsistencies in defining astrocytes, together with the lack of well‐characterized in vitro models, have largely prevented these isolated findings from coalescing into a comprehensive model of astrocyte development. Only recently, with the advent of genome‐wide approaches to monitor cell states, it has become feasible to comprehensively define the molecular identity of astrocytes. Comparing NSCs and different stages of astrocyte development on a global, genome‐wide level has allowed to dissect the nuanced molecular changes, often more quantitative than qualitative, that exist between NSCs and early and late stages of astrocyte development, which is an important step towards identifying the mechanisms driving these changes.

### 4.1 Defining astrocyte identity and development on a molecular level using omics approaches

In recent years, major progress has been made in defining the changing molecular identities of astrocytes along their developmental trajectory. The Barres lab made again pioneering contributions to the field of astrocyte research by providing genome‐wide transcriptional profiles of mouse and human astrocytes (Cahoy et al., 2008; Zhang et al., 2014; Zhang et al., 2016), which led to the identification of new astrocyte markers. They also provided a first global analysis of transcriptional changes during astrocyte development in humans in vivo, comparing by bulk RNA‐Seq an astroglial progenitor stage (obtained from the fetal brain at around gestational week 20) to mature astrocytes obtained from the adult human brain (Zhang et al., 2016). While this study lacked sufficient resolution to distinguish different stages of astrocyte development, it became an important reference resource defining the molecular identity of immature and mature astrocytes in humans. For example, one elegant subsequent study analyzed in detail human astrocyte development in a human organoid model, which they validated against this in vivo reference dataset (Sloan et al., 2017).

Such careful molecular validations of in vitro models are particularly needed when studying astrocytes, since, as already discussed in Section 3.3, it is unclear to what extent in vitro models recapitulate astrocyte differentiation in vivo and what stages in the differentiation trajectory they represent. Among the many studies that use in vitro models of astrocyte development, there is no consensus in the definition of terms such as astrocyte progenitors, immature and mature astrocytes. Some studies use the term “mature astrocytes” in vitro for all cells that are non‐proliferative, for cells with improved functional properties (e.g., increased glutamate uptake) compared to other stages or populations, or even for cells that are just characterized by high GFAP expression. However, immature astrocyte populations that have stopped dividing do exist in vivo (Lattke et al., 2021), indicating that cell cycle exit is not sufficient to define astrocytes as mature. Also, it is usually not possible to determine whether functional properties displayed by astrocytes in vitro are quantitatively comparable to the functional properties of mature astrocytes in vivo, and therefore functional assays can only provide a relative measure of maturation. Most importantly, high GFAP expression levels in vivo indicate astrocyte reactivity rather than maturation, therefore GFAP expression should not be used as a measure of maturity. Thus, it is problematic and potentially misleading to define cultured astrocytes as “mature” using the criteria mentioned above, as it suggests that these populations resemble mature astrocytes found in the adult brain, which is unlikely (see also Section 3.3). Indeed, our recent transcriptomic analysis showed that all murine in vitro astrocyte models examined, including astrocytes differentiated from cultured NSCs using BMP4, primary astrocytes and ESC‐derived “mature” astrocytes, retain immature gene expression profiles compared to astrocytes acutely isolated from the adult brain (Lattke et al., 2021). This suggests that the mechanisms of astrocyte differentiation analyzed in in vitro models only recapitulate early stages of differentiation of NSCs into immature astrocytes, highlighting the necessity to benchmark in vitro models against the in vivo situation.

Recently, single‐cell RNA‐sequencing (scRNA‐Seq) and other genome‐wide approaches have begun to shed light on the distinct stages through which astrocytes develop in vivo, although many uncertainties remain. For example, the exact relationship between the trajectories of the oligodendrocyte and astroglial lineages and to what extent they share precursor stages is not fully understood. While the existence of dedicated oligodendrocyte precursor cells (OPCs or NG2‐glia) is well established in the adult mouse brain, recent single‐cell‐RNA‐Seq studies and transplantation experiments suggest the existence of a single bipotent proliferating progenitor population that might generate both oligodendrocytes and astrocytes in the developing brain (Han et al., 2013; Lattke et al., 2021; Weng et al., 2019). However, histological proliferation and fate mapping analyses suggest that the local proliferation of differentiated astrocytes might also contribute to the expansion of the astrocyte population in the developing cortex (Ge et al., 2012). Interestingly, our recent scRNA‐Seq analysis of postnatal and adult mouse striatal astrocytes suggests that at early postnatal stages, astrocytes progress through multiple immature stages after exiting the cell cycle, before they become fully mature in the adult brain (Lattke et al., 2021), although these immature astrocyte stages remain to be validated and characterized in more detail.

Beside the identification and characterization of developmental stages, the characterization of the local and regional heterogeneity of astrocytes has also been greatly facilitated in recent years by high‐throughput sequencing approaches. Despite long‐recognized morphological differences between adult astrocyte subpopulations, only recently tools have become available to dissect this heterogeneity in more detail. A number of recent studies have identified transcriptional differences between astrocytes from different brain regions (Batiuk et al., 2020; Boisvert et al., 2018; Chai et al., 2017; Zeisel et al., 2018) and cortical layers (Bayraktar et al., 2020; Lanjakornsiripan et al., 2018). Although these differences appear gradual and relatively subtle compared to differences observed between different subtypes of neurons, some studies have shown that molecular differences between astrocyte populations are functionally relevant (Chai et al., 2017; Molofsky et al., 2014; Tsai et al., 2012).

Similar to the transcriptional profile of astrocytes, the epigenomic profile of astrocytes and the changes that occur in this profile during differentiation are getting increasingly well characterized, although this analysis has so far been mainly restricted to early steps of astrocytic differentiation and often to cultured astrocytes. Early studies had already implicated DNA demethylation at the GFAP promoter in the gliogenic switch of embryonic neural progenitors (Takizawa et al., 2001; Teter et al., 1996). More recently, studies using Microarray‐based Integrated Analysis of Methylation by Isoschizomers (MIAMI) and whole‐genome bisulfite sequencing (WGBS) concluded that a similar demethylation of promoters of astrocyte‐specific genes occurs widely prior to astrocyte differentiation (Hatada et al., 2008; Sanosaka et al., 2017). Another study profiled genome‐wide histone modifications in an in vitro model of astrocyte differentiation and showed that the deposition of active chromatin marks (H3K4me1 and H3K27ac) at regulatory elements of astroglial genes preceded the induction of expression of these genes (Tiwari et al., 2018). While these studies focused on epigenetic changes during astrocyte differentiation in vitro and/or during early stages of differentiation in vivo, we have performed Assay for Transposase‐Accessible Chromatin using sequencing (ATAC‐Seq) to characterize chromatin accessibility changes during the maturation of cortical astrocytes in vivo and in an in vitro model (Lattke et al., 2021). We showed that chromatin opening at putative regulatory elements strongly correlates with the induction of expression of mature astrocyte‐specific genes. Overall, the current literature suggests that early changes in DNA methylation and histone modifications, by priming the chromatin for the opening at astrocyte‐specific regulatory elements and subsequent induction of astrocyte‐specific gene expression, define a fundamental astrocyte identity on an epigenetic level.

Further dissection of the molecular changes underlying astrocyte development using novel single‐cell‐ and multi‐omics‐approaches, will deepen our understanding of the process of astrocyte development and facilitate the identification of novel regulators of this process. In the following sections, we will describe functional regulators of astrocyte development that have been identified either by traditional candidate‐based approaches or more recently by systems biology approaches, and we will outline how emerging technologies will allow a more detailed characterization of the molecular mechanisms underlying astrocyte differentiation.

### 4.2 Cell‐intrinsic regulation of astrocyte development

Global analyses of the transcriptional and epigenomic states of astrocytes at different developmental stages have started to draw an outline of the developmental trajectory of astrocytes, but the current knowledge of the mechanisms that shape their molecular identity remains limited. Although a number of studies have identified regulators of this process, it is often not well understood which molecular programs these factors regulate, and at which stages of astrocytic development they act. This makes it difficult to integrate these different findings into a comprehensive model of the regulation of astrocyte differentiation.

Of the different layers of regulation of astrocyte differentiation (Figure 3), the transcription factor repertoire that controls the early stages of fate specification and differentiation of astrocytes has been studied relatively extensively. One of the first transcription factors identified as a regulator of astrogliogenesis is Sox9. Michael Wegner and colleagues reported that ablation of this factor in the developing spinal cord results in reduced numbers of astrocytes and oligodendrocytes (Stolt et al., 2003). Benjamin Deneen and others then identified a core transcription factor network regulating astrocyte differentiation, which is centered around the factors NFIA/B and Sox9. NFIA or NFIB expression in the developing spinal cord was shown to promote astrogliogenesis (Deneen et al., 2006), while Sox9 both induces NFIA expression and acts as a partner of NFIA in a transcription factor complex (Kang et al., 2012). Recently, Zbtb20 was identified as another factor that directly cooperates with NFIB and Sox9 in cortical astrocyte differentiation (Nagao et al., 2016). A few studies have started to elucidate the upstream regulation of this core complex. In particular, PITX1 was recently reported to promote astrocyte differentiation by inducing Sox9 expression (Byun et al., 2020), while Brn2 was shown to be required for the Sox9‐dependent induction of NFIA (Glasgow et al., 2017). A number of studies have begun to investigate the molecular mechanisms through which this complex promotes astrocyte differentiation, although this is still incompletely understood. NFIX and Nfe2l1 have been identified as transcription factors mediating astrocyte differentiation downstream of the NFI‐Sox9 module (Matuzelski et al., 2017; Molofsky et al., 2013), whereas Sox3 and Sox10 have been reported to antagonize the NFI‐Sox9‐dependent induction of astroglial genes (Glasgow et al., 2014; Klum et al., 2018).

Of note, most of the studies cited above have focused on astrocyte development in the spinal cord, and it is currently not clear whether the same mechanisms are fully recapitulated in other regions of the CNS. For example, a recent study found region‐specific functions of NFI factors in the adult brain (Huang et al., 2020), which might also be the case during development.

While the studies discussed above showed that the factors investigated are required for fate specification and differentiation of astrocytes, they did not address in detail to which extent these factors are also involved in the functional maturation of astrocytes during postnatal development. Currently, very little is known of the transcriptional control of astrocyte maturation. In vitro, Runx2 and p53 were reported to promote astrocyte maturation (Li et al., 2019; Tiwari et al., 2018), although as discussed in Section 3.3, it is unclear to which extent the in vitro models used in these studies recapitulate maturation in vivo. In our recent study, we identified four transcription factors, Rorb, Dbx2, Lhx2, and Fezf2, whose expression is induced during astrocyte maturation in vivo, but not in in vitro‐differentiated astrocytes. These four factors induced distinct modules of mature astrocyte‐specific genes when their expression was forced in in vitro‐differentiated astrocytes (Lattke et al., 2021). This study suggests that the maturation of astrocytes is regulated in a modular manner by multiple transcription factors acting independently, which is in contrast to the highly coordinated early differentiation program and may contribute to the diversity and plasticity of astrocyte functions.

Interestingly, before their implication in astrocyte maturation, Rorb, Dbx2, Lhx2, and Fezf2 had been shown to be involved in neuronal subtype specification and/or NPC patterning in embryonic development (Chen et al., 2008; Muralidharan et al., 2017; Oishi et al., 2016; Pierani et al., 1999), indicating that astrocyte differentiation involves an extensive re‐use of transcription factors implicated in other neurodevelopmental processes, and highlighting the context‐dependent functions of these factors. Similarly, the homeobox transcription factor Nkx2.1, which is involved in ventral patterning of NPCs, is also required for the generation of telencephalic astrocytes (Minocha et al., 2017).

Multiple other transcription factors regulate astrocyte differentiation as effectors or modulators of different signaling pathways, such as SMADs, STATs, and Rbpj, activated by BMPs, IL‐6‐family cytokines and Notch ligands, respectively, and will be discussed in the following chapter. Overall, while a number of transcriptional regulators have been identified, many questions remain about the transcriptional control of astrocyte differentiation. For example, how the expression of these factors is regulated, how they cooperate in the differentiation process, and how they regulate their target genes on the genomic level, remains poorly understood. Even less is known of how transcription factors, together with epigenetic regulators, reshape the epigenomic landscape during astrocyte differentiation. NFIA, Atf3, and Runx2 have been shown to promote H3K27 acetylation at their binding sites to increase chromatin accessibility (Tiwari et al., 2018). Similarly, we have shown that forced Rorb and Lhx2 expression in in vitro‐differentiated astrocytes promotes chromatin opening at putative binding sites (Lattke et al., 2021). However, which epigenetic regulators the transcription factors interact with to promote chromatin remodeling, is still unknown. In other studies, a few epigenetic regulators have been implicated in astrocyte differentiation, including the histone acetylase CBP/p300 (Nakashima et al., 1999), the DNA methyltransferase Dnmt1 (Fan et al., 2005), and a complex of MeCP2, SIN3A and HDACs (Cheng et al., 2011). Because of the broad activities of these epigenetic regulators and because they were studied in in vitro models of astrogliogenesis, it remains difficult, however, to draw conclusions on the specific role of epigenetic regulators in astrocyte differentiation in vivo.

### 4.3 Regulation of astrocyte development by extrinsic signals

It has long been recognized that extrinsic signals play an important role in astrocyte differentiation. The first signaling pathways shown to be involved were BMPR‐SMAD‐ and JAK‐STAT‐signaling. The cytokines CNTF, LIF, and CT‐1, which activate JAK‐STAT‐signaling through the common gp130 subunit of their receptors, induce GFAP expression and astrocyte‐like morphology in cultured NPCs, and the inactivation of various components of this pathway has been shown to disrupt astrogliogenesis both in vitro and in vivo (Barnabe‐Heider et al., 2005; Bonni et al., 1997; Koblar et al., 1998). Similarly, BMP receptor signaling induces astrocyte differentiation in vitro, which has been proposed to occur through a common transcription factor complex including SMAD and STAT transcription factors and the coactivator CBP/p300 (Gross et al., 1996; Nakashima et al., 1999). However, BMPs and JAK‐STAT‐inducing cytokines individually promote the differentiation of cultured NPCs into GFAP‐expressing cells that have different morphologies and proliferative and neurogenic potentials (Bonaguidi et al., 2005), suggesting that the two pathways independently promote distinct aspects of astroglial differentiation. This could occur via the mobilization of different downstream effectors, such as the high mobility group nucleosome‐binding factors HMGN1/2/3, which influence the astroglial vs neuronal lineage choice in the developing cortex downstream of JAK–STAT signaling (Nagao et al., 2014), or REST, which appears to promote astrocyte differentiation downstream of BMPs by suppressing neuronal transcriptional programs (Kohyama et al., 2010). Of note, BMP‐SMAD and JAK–STAT signaling, along with proinflammatory NF‐κB signaling, have also been shown to be major inducers of astrocyte reactivity (Anderson et al., 2016; Brambilla et al., 2005; Lattke et al., 2012; Lattke et al., 2017; Okada et al., 2006; Sahni et al., 2010), suggesting that these pathways drive both gliogenic NSCs and mature astrocytes towards an intermediate immature/reactive astrocytic state.

Additional extrinsic signals seem to be required to promote the further maturation of astrocytes, as astrocytes differentiated in vitro with either BMP4 or CNTF, like primary cultures of postnatal astrocytes, fail to upregulate a large subset of mature astrocyte‐specific genes (Lattke et al., 2021). Such maturation signals are likely to include soluble factors provided by other cell types, such as neurons or endothelial cells, and/or signals that depend on cell–cell‐ or cell‐matrix‐interactions, which are all lacking in monolayer astrocyte cultures. Indeed, high density monolayer astrocyte cultures show increased expression of maturation‐specific genes (Li et al., 2019), and astrocytes in three‐dimensional culture systems display more mature transcriptional profiles (Lattke et al., 2021; Sloan et al., 2017).

A contact‐dependent pathway, which has been shown to be critical for different stages of astrocyte differentiation is Notch‐Rbpj‐signaling. Notch1 and Notch3 expression was shown to induce GFAP expression and astrocyte‐like morphology in cultured adult hippocampal neural stem cells (Tanigaki et al., 2001), Hes5 is an important Notch effector gene which promotes astrocyte differentiation in the developing cerebral cortex (Bansod et al., 2017), and in co‐cultures of early NPCs with neurons, NFIA was identified as a Notch target gene promoting astrocytic differentiation (Namihira et al., 2009). The crucial importance of Notch signaling for the establishment and maintenance of astrocyte identity is further highlighted by studies showing that the deletion of the Notch effector Rbpj in vivo promotes the dedifferentiation of subsets of astrocytes into an NSC‐like state (Magnusson et al., 2014; Zamboni et al., 2020). Interestingly, Notch signaling originating from neurons and endothelial cells has also been shown to induce several mature astrocyte genes, including the important glial glutamate transporter GLT‐1/Slc1a2 (Hasel et al., 2017; Lee et al., 2017), suggesting that Notch signaling might also be implicated in the later step of astrocyte maturation.

Another extrinsic factor important for both astrogliogenesis and astrocyte maturation is growth factor signaling. Disruption of the growth factor‐RAF‐MEK‐ERK pathway by deletion of both Mek1 and Mek2 was shown to interfere with gliogenesis in the developing cortex, which could be rescued by overexpressing the downstream ETS family transcription factor Etv5/Erm (Li et al., 2012). Growth factors of the FGF family, including FGF1 and FGF2, have also been shown to induce the expression of mature astrocyte‐specific genes in murine astrocytes in vitro (Lattke et al., 2021) and to promote the functional maturation of human astrocytes (Roybon et al., 2013).

Neuronal activity might be another crucial astrocyte maturation‐promoting signal, which might be mediated by neurotransmitter signaling via the GPCR‐cAMP pathway, as the cAMP analogues forskolin and dibutyryl‐cAMP induce gene expression and morphological and functional alterations associated with astrocyte maturation (Hasel et al., 2017; Stanimirovic et al., 1999). Beside these largely unconnected observations, however, there is still little known of the signals contributing to astrocyte maturation.

Another open question is to what extent the regional and local heterogeneity of astrocytes is determined by extrinsic signals. While studies in both mouse and human models of astrocyte differentiation have demonstrated that astrocyte heterogeneity is in part due to their origin from different, distinctly patterned progenitors (Krencik et al., 2011; Tsai et al., 2012), it is also clear that extrinsic signals are crucially influencing the region‐specific astrocytic phenotypes. For example, it has been shown that manipulating the levels of Shh signaling in the adult brain alters the region‐specific phenotypes of cerebellar and hippocampal astrocytes (Farmer et al., 2016). Furthermore, neuron‐specific deletion of *Dab1*, a factor required for neuronal migration during cortical development, not only inverts the neuronal layers of the cerebral cortex, but also the layer‐specific morphology of astrocytes (Lanjakornsiripan et al., 2018), suggesting that layer‐specific differences in astrocytic phenotypes are controlled by neuron‐derived signals.

## 5 NEW OPPORTUNITIES FROM ADVANCES IN SYSTEMS BIOLOGY

### 5.1 Uncovering epigenomic mechanisms of astrocyte development

Numerous studies have greatly improved our understanding of the global transcriptional changes occurring throughout astrocyte development, but to understand how these changes are controlled, it will be essential to comprehensively characterize the epigenomic changes that accompany this process, as epigenomic remodeling is a major cell‐intrinsic determinant of the permanent changes in gene expression that occur in cellular differentiation processes (Figure 3).

Demethylation of cytosine and guanosine‐rich DNA sequences in promoter regions (CpG islands) is thought to be essential to render NSCs permissive to astrocyte differentiation and allow the activation of promoters of astroglial genes during the gliogenic switch, as already mentioned (Hatada et al., 2008; Sanosaka et al., 2017). Beside promoters, cell‐type‐specific enhancer elements also play an important role in controlling cell‐type‐specific gene expression programs (Long et al., 2016; Perino & Veenstra, 2016). As active enhancers show an open chromatin configuration (Perino & Veenstra, 2016), we performed an ATAC‐Seq analysis to broadly identify candidate enhancers in murine immature and mature astrocytes in vivo. We identified more than 20,000 putative enhancers, of which over 30% lost or acquired chromatin accessibility during astrocyte maturation (Lattke et al., 2021). Changes in accessibility strongly correlated with changes in gene expression, suggesting that enhancer remodeling might drive the regulation of up to 50% of maturation‐induced genes.

However, not all open chromatin regions represent active enhancers, and analyzing epigenetic modifications associated with active enhancers, such as the histone modifications H3K4me1 and H3K27ac, on a global level during astrocyte differentiation, will be important to focus on bona fide candidate enhancers for more detailed functional analyses, and to get insights into how enhancer remodeling during astrocyte differentiation is controlled. Until recently, chromatin immunoprecipitation followed by sequencing (ChIP‐Seq), was the only method allowing genome‐wide analysis of histone modifications. However, this method requires large numbers of cells and is therefore difficult to perform on acutely isolated astrocytes. Therefore, recently developed low‐input alternatives like Cut&Run or Tag&Run (Kaya‐Okur et al., 2019; Skene & Henikoff, 2017) will greatly facilitate epigenomic profiling approaches.

Recent advances in single‐cell sequencing technologies now allow the analysis of various epigenetic modifications in small, heterogenous cell populations isolated from tissues in vivo, for example, DNA‐methylation by single‐cell whole genome bisulfite sequencing (scWGBS) (Farlik et al., 2015), chromatin accessibility by single‐cell ATAC‐Seq (Cusanovich et al., 2015), and histone modifications and transcription factor binding by single‐cell Tag&Run (Kaya‐Okur et al., 2019). Such approaches can even be leveraged for single‐cell‐multi‐omics approaches, such as sci‐CAR (Cao et al., 2018) or scCAT‐seq (Liu et al., 2019), which combine scATAC‐Seq and scRNA‐Seq, or scNMT‐seq (Clark et al., 2018), which combines the analysis of chromatin accessibility, DNA‐methylation and gene expression in individual single cells. This will allow the analysis of remodeling of cell‐type‐specific enhancers at much higher resolution along the astrocytic differentiation trajectory, which will give deeper insights into the mechanisms by which cell‐type‐specific enhancers and the epigenetic landscape are established, and how this determines gene expression changes during astrocyte development.

A major challenge in understanding the function of enhancers is to identify the particular genes that are regulated by defined enhancers, as enhancers and their target genes interact often over considerable distances via promoter‐enhancer loops that are determined by the three‐dimensional organization of the genome. Therefore the frequently used default assignment of an enhancer to the closest transcriptional start site identifies only a fraction of real interactions and generates a large number of false‐positive interactions. New prediction methods have improved the accuracy of enhancer‐gene pairing, such as the activity‐by‐contact (ABC) model (Fulco et al., 2019), which is based on genomic distance and regulatory element “activity”, defined by genome‐wide chromatin accessibility and the profile of the histone mark H3K27ac, two relatively easy‐to‐obtain types of experimental data. Also, various techniques have been developed in recent years to directly experimentally validate promoter‐enhancer interactions through proximity‐ligation followed by sequencing. While “genome‐wide chromatin conformation capture” (Hi‐C) (Lieberman‐Aiden et al., 2009) is an unbiased method able to map all genome‐wide interactions, its suitability to identify individual enhancer‐promoter‐interactions is limited by the extreme complexity of the libraries and high sequencing depth required to achieve sufficient genomic resolution. Therefore, targeted approaches have been developed that have more practicable requirements, such as Proximity Ligation Assisted Chromatin immunoprecipitation followed by Sequencing (PLAC‐Seq) (Fang et al., 2016), which allows to focus on interactions including promoters by using, for example, ChIP for RNA polymerase II to enrich for DNA fragments that include active promoters.

Although techniques are now available to experimentally validate genomic interactions, demonstrating the actual enhancer function of a genomic element requires the establishment of its activity of inducing gene expression, ideally by manipulating the element in its genomic context, for example, by CRISPR/Cas9‐based enhancer activation or inactivation, or alternatively by using plasmid‐based reporter assays, which are easier to perform but lose the advantage of maintaining the genomic context. Both types of assays have traditionally been performed on an individual candidate enhancer basis but have recently been used in pooled screening approaches allowing to assess hundreds of elements in a single experiment (Fulco et al., 2016; Gasperini et al., 2019; Kheradpour et al., 2013). While these novel approaches have not yet been systematically deployed to investigate astrocyte development, they have a huge potential to generate a detailed model of the changes in the epigenetic landscape occurring during astrocyte development and how these changes drive the observed changes in gene expression and astrocyte properties.

### 5.2 Systematic identification of functional regulators

The explosive development of new sequencing technologies in the last two decades has allowed to analyze transcriptional and epigenomic changes occurring during a biological process on a global, genome‐wide level, often leading to the identification of dozens or hundreds of candidate regulators of the process in a single high‐throughput experiment. In contrast, the methods for the functional validation of candidate regulators usually still use traditional time‐ and resource‐consuming genetic approaches, allowing at best the validation of a handful of selected candidates at a time, therefore representing a major bottleneck. However, CRISPR/Cas9‐based technologies now enable flexible genetic manipulations to be performed in a highly scalable manner, which allows the screening of large numbers of candidate regulators in parallel, in particular when combined with sequencing technologies to computationally separate the effects of multiple pooled regulators tested in an individual experiment.

In the original CRISPR/Cas9 system, the Cas9 nuclease can be directed to specific genomic loci by a sequence‐specific short guide RNA (gRNA) to introduce targeted mutations. In the last few years, nuclease‐dead Cas9 mutants (dCas9) have been fused to a variety of transcriptional activator or repressor domains or to epigenetic modifiers, creating a collection of “gRNA‐programmable” transcription or epigenetic factors which can be targeted to any genomic locus of interest with sequence‐specific gRNAs, in order to flexibly activate or inhibit transcription or introduce epigenetic modifications (Nakamura et al., 2021; Pulecio et al., 2017). As the target sequence‐specific motif of a gRNA contains only 20 nucleotides, gRNA generation can be easily scaled up, and large libraries of up to hundreds of thousands of gRNAs have been generated by combining high‐throughput chemical synthesis of individual target recognition elements and pooled cloning. This allows the manipulation of thousands of different genes or regulatory regions in different cells in one pooled experiment. Early studies of this type were restricted to simple readouts related to cell proliferation or cell death, measuring the relative enrichment of individually barcoded gRNA constructs using bulk sequencing (Gilbert et al., 2014). However, in recent years a number of studies have elegantly combined this approach with scRNA‐Seq (e.g., Perturb‐Seq [Dixit et al., 2016]) to detect gRNA barcodes and whole transcriptomes of tens of thousands of individual cells. This allows to analyze global transcriptomic changes induced by many individual manipulations in a single bulk experiment, although the number of cells that need to be sequenced for each perturbation limits the complexity of the libraries to targeted screens of at most a few hundred candidates, as opposed to truly genome‐wide screens only possible with simpler readouts. The methods described above have yet to be applied to study astrocyte development, but they have a huge potential to accelerate the validation of candidate regulators and help move towards a systems biology level of understanding of the molecular mechanisms underlying astrocyte development.

## 6 PERSPECTIVES FOR CLINICAL MEDICINE

Because of the crucial role that they are thought to play in CNS injury and a variety of neurological disorders, astrocytes are receiving increasing attention as potential targets for therapies (Filous & Silver, 2016; He et al., 2020; Peterson & Binder, 2020). As outlined in Section 2.4, in such pathological conditions, astrocytes undergo a process of reactivity or astrogliosis associated with prominent functional changes. While astrocytes normally have multiple roles in maintaining brain homeostasis, and therefore protect the CNS from damage by pathological insults, astrocyte reactivity can impair these homeostatic functions and thus enhance pathological responses. Progress in understanding the molecular mechanisms underlying astrocyte development may point to new therapeutic strategies that manipulate astrocytes to improve their beneficial neuroprotective, neuromodulatory, and pro‐regenerative functions, while minimizing their detrimental properties in pathological conditions. In the following sections, we will outline two promising strategies, respectively, to restore the homeostatic functions of astrocytes in disease by harnessing developmental mechanisms, and to promote brain regeneration by reverting astrocytes to a more plastic immature state.

### 6.1 Restoring homeostasis by promoting astrocyte maturation

In many neurological conditions, the homeostatic support functions of astrocytes are disrupted as a consequence of astrocyte reactivity. This is thought to contribute to the pathogenesis in various diseases, for example, by disrupting the blood brain barrier, or by impairing the uptake of glutamate released at synapses, resulting in neuronal hyperexcitability, epileptic seizures and excitotoxic neurodegeneration. Experimental models suggest that the pathology of some disorders might even be primarily driven by astrocyte defects, for example in Alexander Disease, which is caused by mutations in the GFAP gene (Sosunov et al., 2018), spinocerebellar ataxia 7 (SCA7) (Custer et al., 2006), inflammatory cerebellar ataxias (Lattke et al., 2017), and some forms of epilepsy (Eid et al., 2019; Epi, 2016; Tanaka et al., 1997; Zhou et al., 2019). While for monogenic disorders, the correction of the mutant gene might become a feasible treatment strategy in the foreseeable future, for example, through advances in CRISPR/Cas9‐based technologies, for more complex disorders involving impaired homeostatic astrocyte functions, restoring or enhancing these functions by a more general approach may be a promising strategy to stop or even revert disease progression. One strategy to achieve this could be to reactivate mechanisms that promote the establishment of homeostatic functions during astrocyte development. For example, inducing the expression of mature astrocyte‐specific genes involved in the clearance of glutamate or in the production of the inhibitory neurotransmitter GABA, including the glutamate transporter GLT‐1 (Slc1a2), glutamine synthetase (Glul), or Maob, a key enzyme in astroglial GABA production (Yoon et al., 2014), may reduce seizure susceptibility in congenital or injury‐triggered epilepsy. As already mentioned, we have recently identified Rorb and Fezf2 as regulators of astrocyte maturation, and found that these transcription factors control the expression of glutamine synthetase and Maob (Lattke et al., 2021). Thus, activating Rorb or Fezf2 in astrocytes, for example, by gene therapy approaches, might reduce seizure susceptibility in epilepsy.

### 6.2 Promoting regeneration by reverting astrocyte maturation

Regenerative responses recapitulate many developmental processes. For example, neurogenesis, axon outgrowth and synapse formation are key processes for the formation of neuronal circuits during development, but also for the reconstruction of these circuits after injury. Immature astrocytes have important functions in supporting the different steps of circuit formation during CNS development, but this activity is highly restricted in mature astrocytes (see Section 2). Reactivating immature astrocyte properties might therefore enhance the limited regenerative potential of the CNS, by generating an environment more supportive of axon growth and synapse formation, and possibly even by facilitating the generation of new neurons by enhancing the neurogenic capacity of astrocytes. Such a pro‐regenerative environment could facilitate endogenous repair processes, but could also promote the maturation of new neurons generated by therapeutic neuronal replacement approaches based on NPC transplantation or by reprogramming of endogenous astrocytes or oligodendrocyte precursor cells, which has been achieved by forced expression of various factors, for example, Sox2, Neurod1, or a combination of Ascl1 and Bcl2 (Gascon et al., 2016; Guo et al., 2014; Heinrich et al., 2014).

Reverting astrocyte maturation is possible in principle, as even complete astrocyte dedifferentiation to an NSC‐like state has been reported in different settings (Buffo et al., 2008; Laywell et al., 2000; Magnusson et al., 2014; Nato et al., 2015). However, the mechanisms that control immature astrocyte functions are still not well understood, for example, which signals and transcription factors regulate the underlying transcriptional programs, and which epigenetic barriers might block their reactivation in mature astrocytes. As a first step to identify such mechanisms, we identified in our recent study several signaling pathways and downstream transcriptional effectors that might control immature astrocyte‐specific transcriptional programs, including the Wnt‐LEF/TCF, growth factor‐MAPK‐ETS and inflammatory IKK‐NF‐κB‐signaling pathways (Lattke et al., 2021). Genome‐wide profiling of chromatin modifications during maturation might identify epigenetic barriers that prevent a full reactivation of immature astrocyte‐specific programs, such as repressive histone marks, and might suggest ways to overcome these barriers. Also, novel CRISPR‐based screening approaches, as outlined in Section 5, will allow the systematic discovery of regulators of immature astrocyte‐specific and potentially regeneration‐promoting transcriptional programs and epigenetic barriers preventing their reactivation. These approaches might eventually lead to the identification of novel therapeutic targets to promote CNS regeneration.

## 7 CONCLUSION

As we have outlined here, astrocytes have various essential functions in CNS development and homeostasis. In disorders and injury of the CNS, astrocytes become reactive, which can disrupt their mature homeostatic functions, aggravating such pathologies, but it can also partially reactivate immature developmental support functions, which may facilitate CNS regeneration. Understanding how these different functions are controlled during astrocyte development could therefore lead to new therapeutic strategies for these disorders by restoring homeostatic functions or reactivating pro‐regenerative developmental functions. However, this understanding has been limited by the unique biological and technical challenges posed by astrocytes. The high degree of similarity between neural stem cells and astrocytes complicates the interpretation of previous studies. These studies relied on a variety of insufficiently characterized in vitro models of astrocyte development and on the detection of GFAP, a classical but suboptimal astrocyte marker also expressed in neural stem cells, as main readout for astrocyte differentiation. This has resulted in a lack of consensus on definitions of developmental states of astrocytes and on assays to assess astrocyte development, which has largely prevented the emergence of a comprehensive model of the regulatory mechanisms and transcriptional and epigenetic changes that drive astrocyte differentiation.

We have made the case here that more thorough and systematic investigations are needed to validate and reconcile the conclusions of many individual studies and produce a coherent and convincing model of the mechanisms underlying astrocyte development. We have also argued that advances in modern genome‐wide profiling techniques, including single‐cell‐ and multi‐omics‐approaches, as well as large‐scale CRISPR‐based screens, have an enormous potential to improve our understanding of astrocyte development. Finally, we have outlined how this improved understanding might be harnessed to develop novel therapeutic strategies based on manipulation of the mechanisms regulating astrocyte maturation. This might allow the restoration of mature astrocyte functions disrupted in CNS pathologies to return to homeostasis, and the reactivation of developmental immature astrocyte functions that might support endogenous CNS regeneration or therapeutic neuronal replacement approaches.

## AUTHOR CONTRIBUTIONS

**Michael Lattke:** Conceptualization (equal); funding acquisition (supporting); writing – original draft (lead); writing – review and editing (supporting). **Francois Guillemot:** Conceptualization (equal); funding acquisition (lead); writing – original draft (supporting); writing – review and editing (lead).

## RELATED WIREs ARTICLES

Building blocks of the cerebral cortex: from development to the dish

Developmental dynamics of neurogenesis and gliogenesis in the postnatal mammalian brain in health and disease: Historical and future perspectives

Maintenance and differentiation of neural stem cells

## Data Availability

Data sharing is not applicable to this article as no new data were created or analyzed in this study.
